# New Appliances and Things Medical

**Published:** 1908-06-06

**Authors:** 


### NEW APPLIANCES AND THINGS MEDICAL.

[We shall be glad to receive at our Office, 28 & 29 Southampton Street, Strand, London, W.C., from the manufacturers, specimens of all new preparations and appliances which may be brought out from time to time.]

### A STARCHLESS INFANTS' FOOD.

"Glaxo," a new food for infants, is prepared by Joseph Nathan and Company, Limited, of 88 Gracechurch Street, London, E.C. Unlike many preparations designed for the artificial feeding of infants, it contains no starch in any form, and for that reason alone it deserves the recognition of medical men. The second special feature of this food is its high percentage of fat. This is present when undiluted in as large a proportion as 25 per cent., which is unusual in dry preparations. "Glaxo" is a cream-coloured granular substance, which can readily be prepared in proportions suitable to each individual by the simple addition of hot water. A little shaking of the mixture produces a fluid very like milk in appearance, and containing all the elements of cow's milk modified in such a manner as to resemble the proportions present in human milk. The sugar is present in the form of lactose, and the casein when precipitated produces the fine curd which alone is suitable for infant digestion. "Glaxo" keeps very well in the tins in which it is supplied, and a special measure is provided for the more accurate preparation of the various strengths of the mixture ordered by the medical attendant. The reports which we have received indicate that this substance is digested without difficulty by infants who are intolerant of diluted preparations of cow's milk in any form. It certainly seems worthy of careful trial in the many varieties of gastro-intestinal disorders of hand-fed babies and in the resulting conditions of malnutrition and wasting.

### MALTED TEA.

We have received from the firm of Le Valery and Co., of 11 Queen Victoria Street, London, E.C., a sample of their Malted Tea, which is sold at the price of 2s. per lb. The malt is incorporated with a blend of teas from Ceylon, Darjeeling, and Travancore, and the resulting mixture produces on infusion a beverage of mild and pleasant taste without astringency. The addition of the malt does not impair the aroma or flavour, while it appears to add to the digestibility of the infusion. The amount of tannin after five minutes' infusion is very considerably less in this malted tea than in the case of Ceylon tea, and rather less than in the usual varieties of China tea, and on this account we have found the preparation well tolerated by dyspeptics. The price is, of course, lower than that of high-class China teas, such as are usually recommended for patients with weak digestions, and this factor, together with the low degree of astringency, should secure for the malted tea the attention of the medical profession. The proportion of theine is not reduced to a degree corresponding with the low proportion of tannin.

